# High Level of Pyrethroid Resistance in an *Anopheles funestus* Population of the Chokwe District in Mozambique

**DOI:** 10.1371/journal.pone.0011010

**Published:** 2010-06-08

**Authors:** Nelson Cuamba, John C. Morgan, Helen Irving, Andrew Steven, Charles S. Wondji

**Affiliations:** 1 National Institute of Health, Maputo, Mozambique; 2 Vector Group, Liverpool School of Tropical Medicine, Liverpool, United Kingdom; Natural History Museum of Denmark, Denmark

## Abstract

**Background:**

Although *Anopheles funestus* is difficult to rear, it is crucial to analyse field populations of this malaria vector in order to successfully characterise mechanisms of insecticide resistance observed in this species in Africa. In this study we carried out a large-scale field collection and rearing of *An. funestus* from Mozambique in order to analyse its susceptibility status to insecticides and to broadly characterise the main resistance mechanisms involved in natural populations.

**Methodology/Principal Findings:**

3,000 F_1_ adults were obtained through larval rearing. WHO susceptibility assays indicated a very high resistance to pyrethroids with no mortality recorded after 1h30min exposure and less than 50% mortality at 3h30min. Resistance to the carbamate, bendiocarb was also noted, with 70% mortality after 1h exposure. In contrast, no DDT resistance was observed, indicating that no *kdr-type* resistance was involved. The sequencing of the acetylcholinesterase gene indicated the absence of the G119S and F455W mutations associated with carbamate and organophosphate resistance. This could explain the absence of malathion resistance in this population. Both biochemical assays and quantitative PCR implicated up-regulated P450 genes in pyrethroid resistance, with GSTs playing a secondary role. The carbamate resistance observed in this population is probably conferred by the observed altered *AChE* with esterases also involved.

**Conclusion/Significance:**

The high level of pyrethroid resistance in this population despite the cessation of pyrethroid use for IRS in 1999 is a serious concern for resistance management strategies such as rotational use of insecticides. As DDT has now been re-introduced for IRS, susceptibility to DDT needs to be closely monitored to prevent the appearance and spread of resistance to this insecticide.

## Introduction

The mosquito *Anopheles funestus* is a major vector of malaria throughout much of sub-Saharan Africa, but because it is a relatively intractable species to work with, it has only recently started to receive the scientific attention such an important public health pest deserves. The highly anthropophilic and endophilic behaviours of this mosquito make it an efficient vector of malaria, and in many places, parasite infection rates of *An. funestus* exceed those of *An. gambiae* [1].

Pyrethroid resistance has been reported in populations of this species in southern Africa [2], [3], [4]. In Mozambique, resistance to pyrethroid has been observed in the Southern part of the country since 2000 [2] and elevated P450 monooxygenases were implicated [2], [3]. Recent effort to further elucidate mechanisms of pyrethroid resistance in *An. funestus* has used the laboratory selected resistant strain FUMOZ-R from Mozambique [5], [6], [7]. These studies have confirmed the role of P450 genes in conferring pyrethroid resistance, but the specific P450s involved are still not fully characterised. This characterisation is necessary for the identification of specific markers that could be used to design diagnostic assays to detect and monitor this resistance. For such detailed characterisation and identification of causative mutations, it is important to define the natural genetic variation within field populations of *An. funestus* rather than rely on a laboratory strain. One of the obstacles in working with field samples of *An. funestus* is the difficulty in rearing F_1_ material from field collected mosquitoes of this species to obtain the large numbers needed for an extensive resistance characterisation including WHO susceptibility tests, biochemical, genetic and molecular assays. Only few colonies of *An. funestus* have so far been established in the laboratory [8] and there is a need to improve the rearing of field samples of this species in order to facilitate genetic and molecular studies such as QTL mapping, microarray analysis.

As a first step in a broad study to characterise pyrethroid resistance in field populations of *An. funestus* in Africa, we report here a large-scale field collection and rearing of an *An. funestus* population from Chokwe district in Mozambique in order to analyse its susceptibility status to insecticides and to broadly characterise the main operating resistance mechanisms. The level of pyrethroid resistance in this *An. funestus* population from Mozambique was far above that reported to date.

## Materials and Methods

### Area of study and mosquito collection

Blood fed *An. funestus* adult females resting indoor were collected in houses between 06 and 12AM in Tihuquine (Chokwe District) located 20 km from the town of Chokwe (24°33′37″S, 33°1′20″E) in southern Mozambique. The collection was carried out over two week period in February 2009 during the raining season. Mosquitoes were collected using aspirators and torches and transported to the insectary of the entomological laboratory of the National Institute of Health in Maputo. 130 females were set up in individual oviposition cups and hundreds were set up in common cages for collective egg laying at 26+/−2°C and 75–85% relative humidity. Eggs laid were sent in batches on wet filter paper to the Liverpool School of Tropical Medicine (LSTM) where they were allowed to hatch in small cup and latter transferred to larvae bowl for rearing. Larvae were fed abundantly with Tetra Tetramin™ baby fish food every day. We experimentally used distilled and mineral water to identify the best water for larval rearing. Water in each larval bowl was changed every two days to reduce mortality due to poor water quality.

### PCR-species identification

All females used for individual oviposition and a subset used for the mixed cage were morphologically identified as belonging to the funestus group according to the key [9]. A PCR was carried out using the protocol of [10] to confirm that collected females are *An. funestus s.s*.

### Insecticide susceptibility assays

Insecticide susceptibility assays were carried out using 1–3 day-old F_1_ adults from both single families and mass-reared mosquitoes following the WHO protocol [11]. Around 20–25 mosquitoes per tube were exposed to insecticide-impregnated filter paper for 1h and then transferred to a clean holding tube supplied with 10% sugar and mortality was determined after 24h. We tested the following insecticides: the pyrethroids permethrin (0.75%), deltamethrin (0.05%), lambda-cyhalothrin (0.05%); the carbamate bendiocarb (0.01); the organophosphate malathion (5%) and DDT.

### Biochemical assay

Biochemical assays based on the method described by [12] were carried using 50 adults aged between 1 to 3 days from the Tihuquine mixed F_1_ mosquito set. The same number of *An. gambiae* mosquitoes from the Kisumu strain was used as the susceptible control sample since no live susceptible strain from *An. funestus* was available, as similarly previously done [3], [13]. The following enzyme assays were carried out: glutathione S-transferase (GST), altered acetylcholinesterase (*AChE*), esterase assays (pNPA and α- and β- naphthyl acetate), monooxygenase (P450). We used a two-sample *t*-test to compare the results of the biochemical assays between the susceptible strain (Kisumu) and the field samples from Tihuquine following an adjustment for total protein content.

### Acetylcholinesterase (*AChE*) sequencing

A fragment of the acetylcholinesterase gene (*AChE*) (Accession number: DQ534435), spanning the G119S and F455W mutations previously associated with carbamate resistance [14], [15], was amplified and sequenced in carbamate resistant and susceptible mosquitoes from Tihuquine in order to detect these two mutations or others associated with carbamate resistance. DNA was extracted using the LIVAK method [16]. The following primers were used for the PCR amplification: *AChE* Forward CCA CTG TCG GAG GAC TGT CT and *AChE* Reverse CGT TAA CGT ACG GGT CGA GT. The PCR was carried out using 10 pmol of each primers and 30ng gDNA as template in 25µl reactions containing 1× Kapa Taq buffer, 0.2mM dNTPs, 1.5mM MgCl_2_, 1U Kapa Taq (Kapa biosystems). The cycle parameters were: 1 cycle at 95°C for 5 min; 35 cycles of 94°C for 30s, 57°C for 30s and elongation at 72°C for 1min; followed by 1 cycle at 72°C for 10 min.

### Transcription profiling of some P450 genes

We carried out a quantitative PCR (qPCR) analysis of mosquitoes for two copies of two P450 genes (*CYP6P9a*, *CYP6P9b*, *CYP6P4a*, *CYP6P4b*) previously found to be associated with pyrethroid resistance in FUMOZ-R strain [6] to see if they were also over-expressed in these field samples. The GeXP genetic analysis system from Beckman and Coulter was used according to the protocol in [6]. RNA was extracted using the Picopure RNA isolation kit (Arkturis) from three batches of 10 females and 3 batches of 10 males from a random sampling of adult *An. funestus* from the village of Tihuquine. The same was done for the susceptible laboratory FANG strain (originating from Angola [8]) and KELA, a field susceptible strain from Mali. Both FANG and KELA were stored in RNAlater and for this reason were not used for the biochemical assays. The following primers were used: CYP6P9a/b Forward AGGTGACACT ATAGAATACA ATGTGATAAA CGAAACACTT CGCAA (common to both CYP6P9 copies and spanning the intron); CYP6P9a Reverse *GTACGACTCA CTATAGGGA*C TTTATTATAG ATTGGAAGTA TCTCA (expected product at 490bp); CYP6P9b Reverse *GTACGACTCA CTATAGGGA*C TACAAAAACC CCTTCCGCTG CACC (expected product at 504bp); CYP6P4a/b Forward *AGGTGACACT ATAGAATA*TA ATGTGATCAA CGAAACTCTA CGCAAAT (common to both CYP6P9 copies and spanning the unique intron); CYP6P4a Reverse *GTACGACTCA CTATAGGGA* CGTTTCCATG GAATTACATT TTCTG (expected product at 449bp); CYP6P4b Reverse *GTACGACTCA CTATAGGGA*A CAATCATTAT ACCACACATC TGAC (expected product at 539bp).

## Results

### Mosquito collection

A total of 2000 indoor-resting *Anopheles* mosquitoes were collected inside houses in the village of Tihuquine over a period of 2 weeks in February 2009. Around 1500 were morphologically identified as belonging to the *An. funestus* group and ∼500 were identified as belonging to the *An. gambiae* complex. Most of these mosquitoes were blood-fed, half gravid or gravid.

### Mosquito rearing

From the 130 oviposition cups set up with individual gravid *An. funestus* females, 65 produced egg batches. Females from the common cage also laid several egg batches. These eggs were successfully transported to Liverpool where twenty of the best individual egg batches and the mass-reared eggs batches were successfully reared to the adult stage. Larvae reared in mineral water grew quicker and look fitter than those reared in distilled water. As a consequence, pupae were obtained 2 days earlier from mineral water bowls than for distilled water bowls. The rearing time from egg to adult was 16 days, well below the 36 days mentioned for the rearing of the FUMOZ and FANG strains [8]. More than 3000 F_1_ adult mosquitoes were obtained from this field sample rearing.

### Susceptibility tests

There was a very high level of pyrethroid resistance in the *An. funestus* population of Tihuquine measured by WHO bioassays. No mortality was recorded when mosquitoes (both males and females) were exposed to 0.75% permethrin, 0.05% deltamethrin or 0.05% lambda-cyhalothrin for 1h and 1h30 respectively (Table 1). Because of this high level of resistance the quality of the WHO papers used for the tests were checked for insecticide concentration. The same WHO papers tested on the *An. gambiae* pyrethroid resistant laboratory strain RSP. A level of 85% mortality was obtained for this strain as expected. Additional *An. funestus* samples were tested at exposure times of 2h30 or 3h30 to further analyse the mortality rate. Only 20% mortality was observed at 2h30 for both 0.05% deltamethrin and 0.05% lambda-cyhalothrin while 35% mortality was observed for 0.75% permethrin. The mortality rate increased to 30% for 0.05% deltamethrin and to 45% for 0.75% permethrin when mosquitoes were exposed for 3h30 (Table 1). This resistance level is remarkably high since previous published data showed that *An. funestus* from Chokwe was pyrethroid susceptible in 2000 and 2002, and was initially selected in 2006 with only a mortality of 84% after 1h exposure to 0.05% lambda-cyhalothrin [3], [13].

**Table 1:** WHO susceptibility test results on 1–3 day old F_1_ *An. funestus* from Tihuquine.

	Total number of mosquitoes tested	% Mortality			
		1h	1h30	2h30	3h30
Permethrin (0.75%)	440	0±0	0±0	35±4.0	45±5.3
Deltamethrin (0.05%)	205	0±0	0±0	20±4.1	30±2.7
Lambda-cyhalothrin (0.05%)	145	0±0	0±0	20±3.5	nd
Bendiocarb (0.01%)	250	70±3.2	80±2.5	nd	nd
DDT (4%)	100	100±0	nd	nd	nd
Malathion (5%)	75	100±0	nd	nd	nd

nd, not done.

A mortality rate of 70% was observed for 0.01% bendiocarb at 1h exposure indicating carbamate resistance in *An. funestus* from Tihuquine. This mortality rate increased to 80% when mosquitoes were exposed at 1h30min. This resistance level is higher than that observed in the area between 2000 and 2002 (no resistance) and between 2002 and 2006 (96% mortality) [3], [13] suggesting an increase of the resistance with time.

No resistance was observed for DDT and malathion (organophosphate), with 100% mortality at 1h exposure for both 4% DDT and 5% malathion. This result is similar to that observed previously in the area [3], [13].

### Biochemical assay results

**Figure 1:**
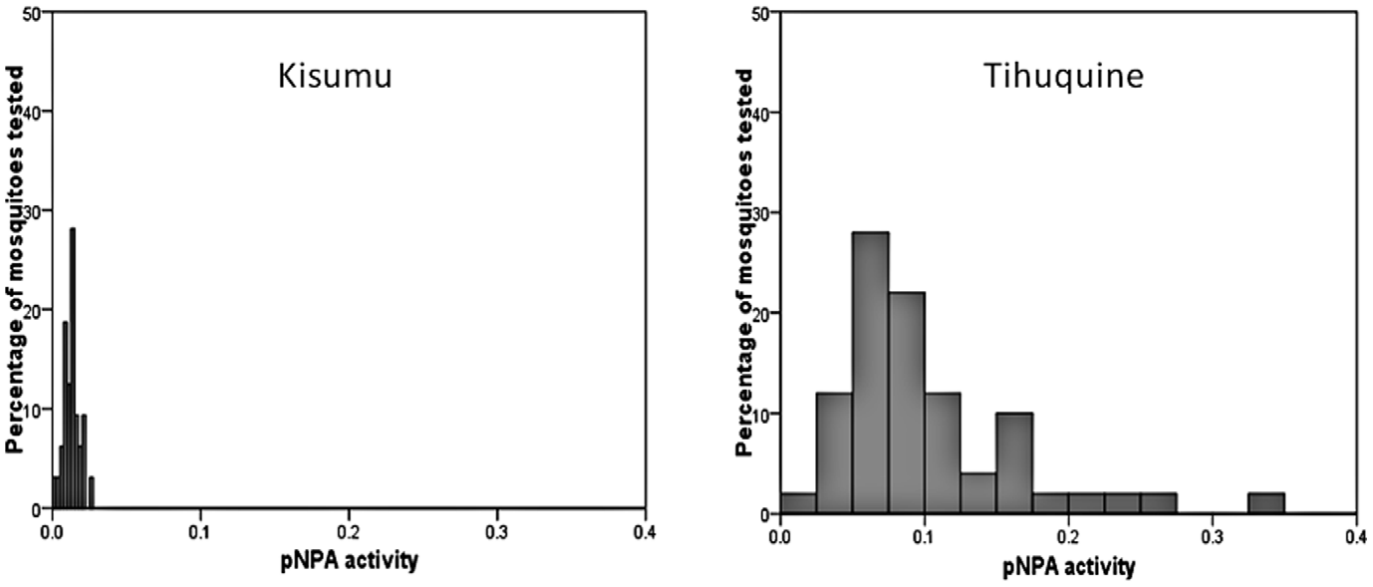
Esterase activity. Range of esterase activity with the substrate *p*-nitrophenyl acetate in the *An. funestus* population of Tihuquine compared with *An. gambiae* Kisumu reference strain.

A significant increase in esterase activity was observed with the substrate pNPA in the Tihuquine population compared to the Kisumu susceptible strain (P<0.001). The pNPA activity ranged from 0.02 to 0.33 with an average of 0.101 for Tihuquine while it ranged from 0.004 to 0.027 with an average of 0.0126 for the susceptible strain (Figure 1, Table 2). No significant difference was observed with both α and β-Naphthyl acetate substrates (Table 2).

**Table 2:** Comparisons of average values for a range of biochemical assays between F_1_ adult progeny *An. funestus* from Tihuquine populations and the *An. gambiae* Kisumu insecticide-susceptible reference strain.

	Chokwe		Kisumu
	Average	*P* value	
pNPA	0.101	0.001	0.0126
α-Naphthyl acetate	3.52 10^−05^		3.68 10^−05^
β-Naphthyl acetate	5.9 10^−05^		5.03 10^−05^
P450	0.009	0.02	0.004
GST	0.336	0.001	0.0161
*AChE*	45.2	0.001	67.4

α- naphthyl acetate: mMoles α-naphthol produced/min/mg protein.

β- naphthyl acetate: mMoles β-naphthol produced/min/mg protein.

p450: Equivalent units of cytochrome p450. GST: GST activity.

*AChE*: average percentage inhibition of AChE by propoxur.

**Figure 2:**
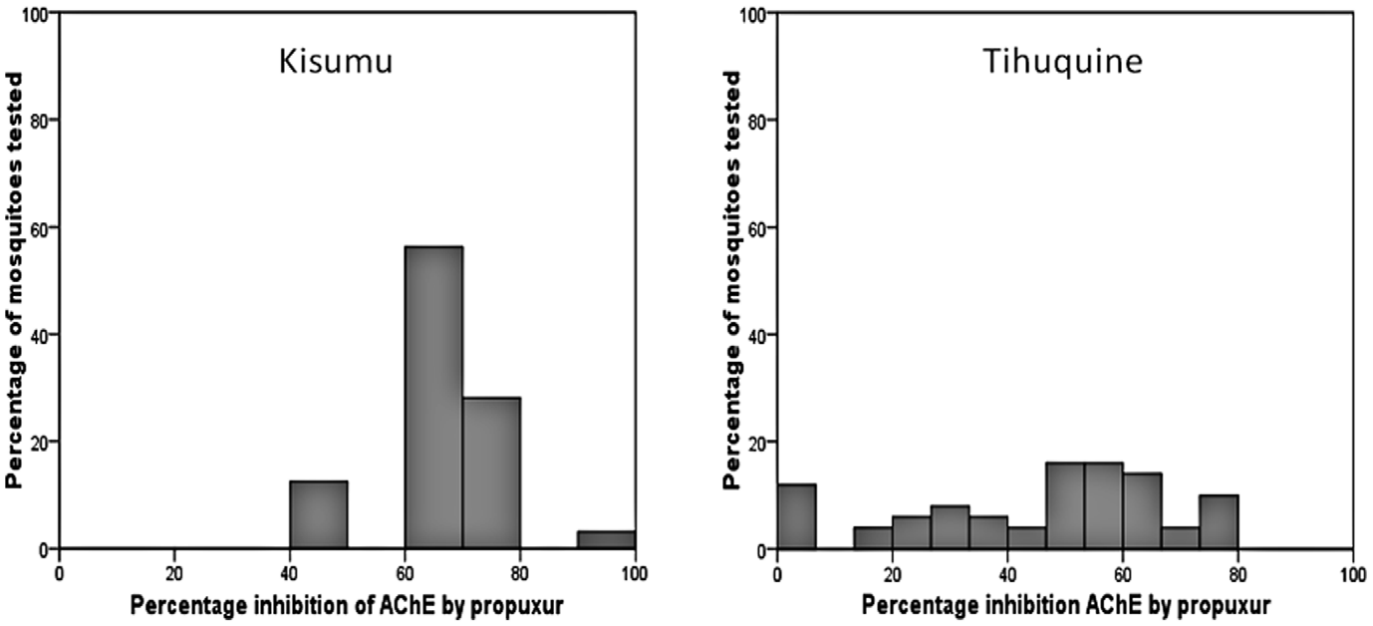
Acetylcholinesterase inhibition rate by propuxur. Acetylcholinesterase inhibition ranges in the *An. funestus* from Tihuquine compared to the *An. gambiae* insecticide-susceptible Kisumu reference strain.

The profiles of *AChE* inhibition rates by propoxur were significantly different between the Tihuquine population and the Kisumu strain (P<0.001). *AChE* inhibition rates by propoxur ranged from 40 to 100% (Figure 2) in the Kisumu susceptible strain with an average of 67.4% (Table 2). In contrast, the range of *AChE* inhibition by propoxur for the *An. funestus* population of Tihuquine was 0 to 80% with an average of 45.2%.

**Figure 3:**
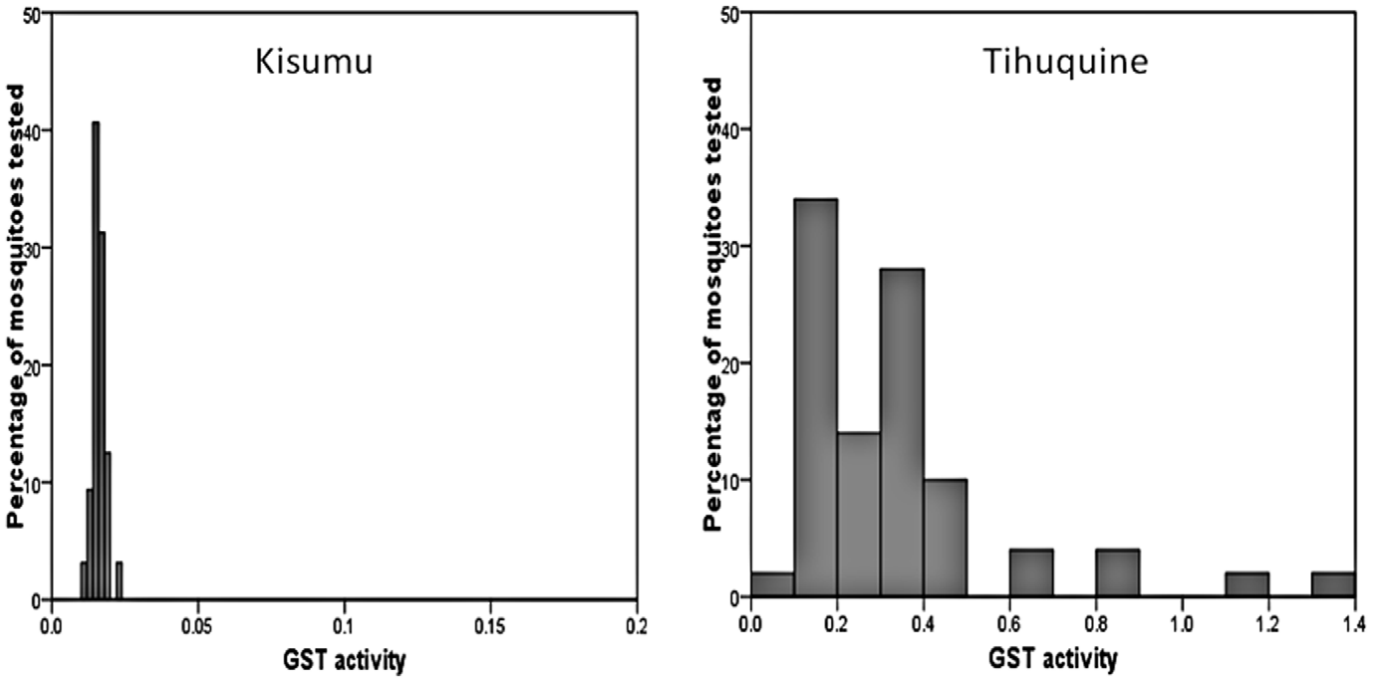
GST activity. Range of GST activity in the *An. funestus* population of Tihuquine compared with *An. gambiae* Kisumu reference strain.

A significant increased in the level of GST activity was observed in the Tihuquine populations compared to the susceptible Kisumu strain (P<0.001). The GST activity in the Tihuquine population ranged from 0 to 1.40 with an average of 0.336 while it ranged from 0 to 0.025 in the Kisumu strain with an average of 0.016 (Figure 3).

**Figure 4:**
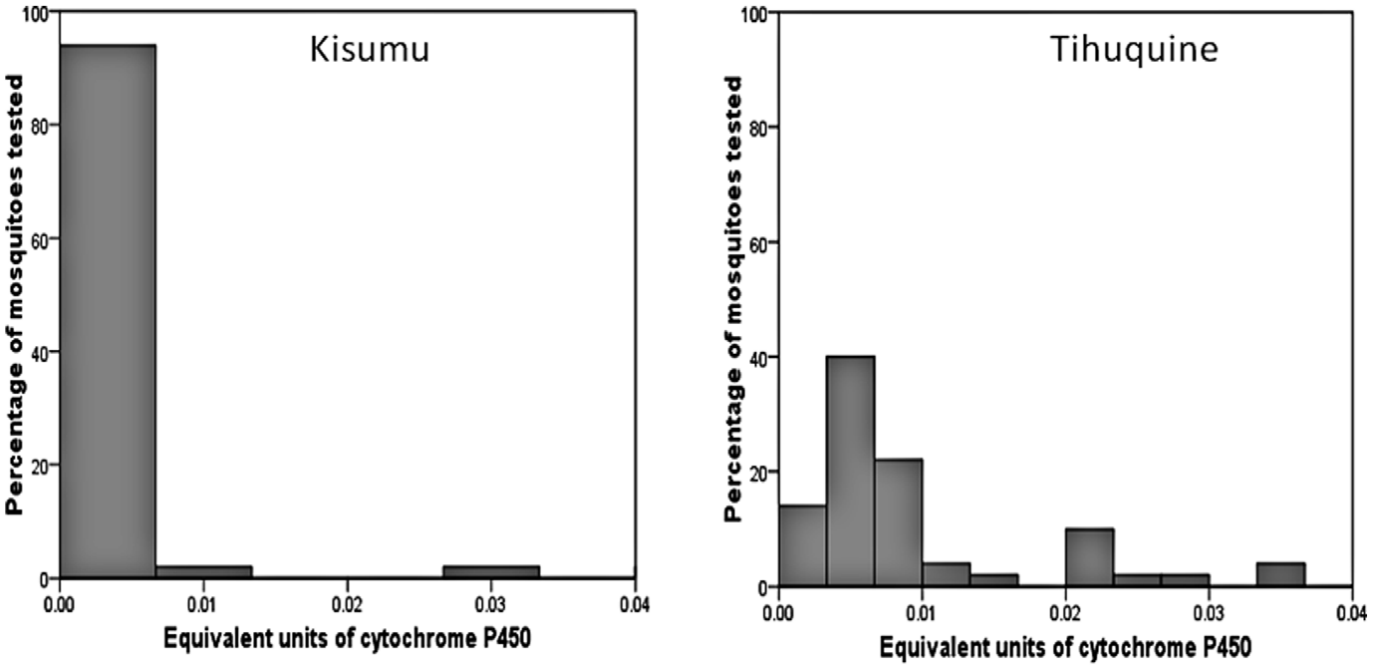
Monooxygenase activity. Estimated levels of cytochrome P450s (representing monooxygenase activity) in the *An. funestus* population of Tihuquine compared with *An. gambiae* Kisumu reference strain.

Increased level of monooxygenases (2.25 fold) were detected in the Tihuquine population compared to the Kisumu strain (P<0.05) (Figure 4) indicating that a metabolic resistance mechanism through the cytochrome P450 genes is operating in the Tihuquine population.

### *AChE* gene sequencing

We sequenced a 1290 bp fragment of the *AChE* gene in twenty mosquitoes, fifteen from Mozambique (8 bendiocarb resistant and 7 susceptible) and five from Cameroon (fully susceptible to all insecticides (C. Wondji, Unpublished). The G119S mutation that confers carbamate and organophosphate resistance in *An. gambiae* and *Culex quinquefasciatus* [15] was not observed in these samples. The glycine amino acid codon is GGA in *An. funestus* not GGC as seen in *An. gambiae*. This implies that two independent mutations are needed to change glycine to serine in *An. funestus*. The F455W mutation observed in *Cx tritaeniorhynchus* [14] and *Cx gelidus* (H. Wilkins, unpublished data) was not observed in this study. Here again instead of the TTC codon, as in *An. gambiae*, it is the TTT codon that encodes for the phenylalanine (F). Two independent mutations are also needed at this codon to obtain the TGG codon for tryptophan (W) amino acid as seen in *Cx tritaeniorhynchus* [14]. Several indels (from 2 to 45 nucleotides) are observed in the introns without a correlation with resistance pattern or geographic location, as they were observed in both Cameroon and Mozambique samples. A high level of synonymous polymorphism was also observed in the *AChE* gene although not associated with resistance. However no amino acid change was observed in the entire sequence fragment. In general only 3 amino acid changes were observed when compared to the *An. gambiae* sequence.

### Transcription profiling of some P450 genes

**Figure 5:**
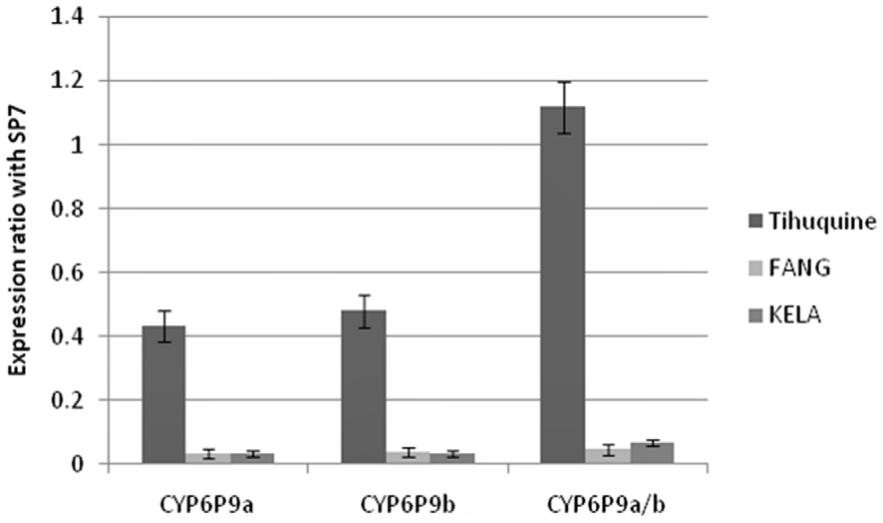
Relative expression levels of candidate genes using quantitative RT-PCR. Comparison of the patterns of gene expression of each copy of the duplicated *CYP6P9* (*CYP6P9a* and *CYP6P9b*) and when both are amplified together (*CYP6P9a/b*) between the resistant field sample of Tihuquine, the laboratory susceptible strain FANG and KELA, a susceptible field sample from Mali. The normalised expression ratio of each gene against SP7 gene is represented on the vectical axis.

Due to the increased level of monooxygenases observed in the biochemical assay the expression levels of two duplicated P450 genes associated with pyrethroid resistance in FUMOZ-R [6] were analysed in the Tihuquine sample. The quantitative amplification of the two copies of *CYP6P9* and *CYP6P4* showed that only *CYP6P9* was over expressed in the Tihuquine population (Figure 5). *CYP6P9a*, observed at 490bp and *CYP6P9b* at 504bp were both over-expressed in female and male samples. When compared to the *An. funestus* laboratory susceptible strain FANG and the field susceptible sample KELA from Mali, *CYP6P9b is* consistently expressed at a higher ratio than CYP6P9a with respectively a 15-fold and 12-fold over-expression. This confirms that the two copies of *CYP6P9* are transcribed and functional as seen also in other strains of *An. funestus* such as FUMOZ-R (C. Wondji, unpublished data).

## Discussion

*An. funestus* is notoriously difficult to colonize and larvae collection is difficult. For these reason few studies have been carried out on the insecticide susceptibility status of field populations of this important malaria vector in Africa. In this study we collected and reared *An. funestus* to the F_1_ generation in order to analyse the susceptibility status of a population of this species to a wide range of insecticides and to broadly characterise the main resistance mechanisms involved.

We successfully obtained eggs from individual females and mass-reared females. Abundant eggs were laid by individual females which are appropriate for genetic studies, such as QTL mapping of phenotypic traits in field population, where family samples at different generations are needed. This abundant egg laying also allowed eggs to be shipped by mail to a better equipped insectary, where conditions were optimised for larval rearing to increase the number of adults obtained.

Overall larval rearing was very successful with a low mortality rate. This could have been influenced by the feeding and the frequent change of the water. A shorter time for larval rearing was observed in this study compared to previous studies [8]. Water quality was a factor influencing the rearing since larvae grew better in mineral water than in distilled water. Hence, natural water (from wells or natural breeding sites) should be used whenever possible for optimum larval rearing.

The pyrethroid resistance level exhibited by the *An. funestus* population of Tihuquine is remarkably high. Such resistance level has not previously been reported in field populations of *An. funestus*. When compared to previously published data, there is a sharp increase in the resistance level of *An. funestus* from Chokwe. Indeed, no pyrethroid or carbamate resistance was noted in Chokwe district (where Tihuquine is located) prior to 2002, and a low resistance level was noted between 2002 and 2006 [3], [13]. The fact that no mortality was observed at 90 min exposure for both males and females for all three pyrethroids tested constitutes a very sharp increase in the resistance level. This finding indicates that the resistance front in *An. funestus* populations in Mozambique is expanding to areas where this species was once fully susceptible. This should be taken into consideration by the Mozambican malaria control program in its resistance management plans. In other hand, another challenge is to explain why such a high resistance level has been selected in Tihuquine when pyrethroids are not currently used by the Mozambican National Malaria Control Program (MNMCP) for its IRS program. Indeed DDT was reintroduced into Mozambique's IRS programme in 2006 and is increasingly becoming the main insecticide used for malaria vector control in Mozambique. The selection of DDT as the insecticide of choice in Mozambique was evidence-based, taking account of the susceptibility of *Anopheles funestus* to all available insecticide choices. Previously lambda-cyhalothrin replaced DDT in Mozambique in 1993. However, resistance appeared quickly to this insecticide and, in 2000, the pyrethroid was phased out and the carbamate bendiocarb introduced. But because of resistance to bendiocarb, DDT was re-introduced in 2006 [13]. Therefore the selection pressure due to pyrethroids used in IRS programs was expected to have significantly reduced. The increased resistance levels observed in this study suggest that the source of this selection pressure is not from IRS alone but perhaps from the use of pesticides in the agriculture. Indeed Tihuquine is surrounded by ricefield where insecticides are use for pest control. No program of impregnated bed nets is currently implemented in this village to explain such high resistance. It is important to clearly identify the main factor driving such a high resistance, because it could spread rapidly through gene flow to other populations particularly in northern Mozambique were resistance is still very low [3].

The fact that no DDT resistance was observed in this population of Tihuquine excludes the presence of the *kdr* mutation in this population, since it confers cross-resistance to pyrethroids and DDT. Therefore the mechanism of pyrethroid resistance operating in this population is probably caused by a metabolic resistance mechanism as shown by the biochemical assays and the qPCR results in this study.

The increase level of P450s observed in the Tihuquine sample suggests that cytochrome P450s play a major role in conferring pyrethroid resistance, as previously observed in other field populations in Mozambique [2], [3], [13]. The over-expression of both copies of *CYP6P9* observed by qPCR in Tihuquine also suggests that the main mechanism of pyrethroid resistance in this population is elevated levels of P450 enzymes, as also seen in the FUMOZ-R resistant laboratory strain [5], [6]. It has been suggested that these elevated P450s also confer a cross-resistance to carbamates [2]. This could also partly confer the carbamate resistance in Tihuquine. This is particularly likely if the pyrethroid resistance results from a change in a P450 regulatory region, rather than a mutation in a single P450 structural gene. In this case several P450s could be up-regulated, which could produce cross-resistance to several insecticide classes including carbamates.

The significantly elevated level of GST activity seen in the Tihuquine samples (as seen previously in field population in Mozambique [3]) also suggests that GSTs may act as secondary detoxification agent in this *An. funestus* populations as suggested before [3]. This is supported by the recent transcriptome sequencing results of two *An. funestus* strains where a significantly higher copy of *GSTe2* was observed in the resistant strain compared to the susceptible (Gregory et al submitted).

The significant difference observed in the *AChE* inhibition rates by propoxur between the Tihuquine population and Kisumu could be seen as if an altered *AChE* mechanism was present in Tihuquine. This mechanism is known to confer cross resistance between organophosphates and carbamates. However, we did not see such cross resistance in Tihuquine with full susceptibility observed to malathion, an organophosphate. Additionally, the sequencing of the *AChE* gene did not reveal any of the target site mutations (G119S or F455W) associated with altered *AChE* as commonly seen in other mosquito species such *An. gambiae* or *Cx quinquefasciatus*. Therefore, the *AChE* inhibition rates by propoxur observed in the Tihuquine population is not sufficient to prove the presence of an altered *AChE* in Tihuquine. The significant difference observed between the *AChE* inhibition rates of the two samples could be due to some artefacts such as: i) the fact that the reference susceptible strain belonged to another species (*An. gambiae*) and that inhibition constants for propoxur could be different between both species; ii) the fact that biochemical assays were done on total extract of mosquitoes. In that case and since increase activities were demonstrated for esterases and oxidases, there is the possibility that a part of propoxur was hydrolysed or sequestered by these enzymes and that the propoxur concentration efficiently available to inhibit the *AChE* target site was significantly decreased. The carbamate resistance in Tihuquine could be conferred by P450s potentially with the possibility of a cross-resistance between pyrethroids and carbamate as already suggested by [2].

Although the biochemical assays have provided some indications of the potential mechanisms involved in the pyrethroid and carbamate resistances in Tihuquine, more remain to be done to fully elucidate these mechanisms. Samples generated from this field collection are being used presently for more molecular and genetics analysis such as the study of the gene expression pattern of the detoxification genes by microarray or the genetic mapping of genes conferring these resistances using family samples generated from this collection.

### Conclusion

The high level of pyrethroid resistance observed in Tihuquine is a big challenge for resistance management strategies such as insecticide rotation, as applied in Mozambique. Although this study covers only one location, it is likely that due to gene flow, that this resistance has spread in southern Mozambique where resistance is already well reported. The aim of insecticide rotational strategies is to reduce the selection pressure caused by an insecticide so that it can be reused in future. This will not be possible for pyrethroids as pyrethroid and carbamate resistance are still increasing. However, because there is still full DDT susceptibility in *An. funestus*, control of this species is still possible. In this setting the appearance of DDT resistance as seen in *An. funestus* populations of West Africa [17] will have a devastating effect on the control of this species in Mozambique. Therefore, a permanent monitoring of the DDT susceptibility status of *An. funestus* populations in Mozambique is crucial to ensure a successful control of this species.
